# Transcranial magnetic stimulation mapping of the motor cortex: comparison of five estimation algorithms

**DOI:** 10.3389/fnins.2023.1301075

**Published:** 2023-12-07

**Authors:** Yuanyuan Chen, Yihan Jiang, Zong Zhang, Zheng Li, Chaozhe Zhu

**Affiliations:** ^1^State Key Laboratory of Cognitive Neuroscience and Learning, Beijing Normal University, Beijing, China; ^2^Center for the Cognitive Science of Language, Beijing Language and Culture University, Beijing, China; ^3^Center for Cognition and Neuroergonomics, State Key Laboratory of Cognitive Neuroscience and Learning, Beijing Normal University Zhuhai, Zhuhai, China; ^4^IDG/McGovern Institute for Brain Research, Beijing Normal University, Beijing, China; ^5^Center for Collaboration and Innovation in Brain and Learning Sciences, Beijing Normal University, Beijing, China

**Keywords:** transcranial magnetic stimulation, TMS motor mapping, estimation algorithm, electric field modeling, functional magnetic resonance imaging

## Abstract

**Background:**

There are currently five different kinds of transcranial magnetic stimulation (TMS) motor mapping algorithms available, from ordinary point-based algorithms to advanced field-based algorithms. However, there have been only a limited number of comparison studies conducted, and they have not yet examined all of the currently available algorithms. This deficiency impedes the judicious selection of algorithms for application in both clinical and basic neuroscience, and hinders the potential promotion of a potential superior algorithm. Considering the influence of algorithm complexity, further investigation is needed to examine the differences between fMRI peaks and TMS cortical hotspots that were identified previously.

**Methods:**

Twelve healthy participants underwent TMS motor mapping and a finger-tapping task during fMRI. The motor cortex TMS mapping results were estimated by five algorithms, and fMRI activation results were obtained. For each algorithm, the prediction error was defined as the distance between the measured scalp hotspot and optimized coil position, which was determined by the maximum electric field strength in the estimated motor cortex. Additionally, the study identified the minimum number of stimuli required for stable mapping. Finally, the location difference between the TMS mapping cortical hotspot and the fMRI activation peak was analyzed.

**Results:**

The projection yielded the lowest prediction error (5.27 ± 4.24 mm) among the point-based algorithms and the association algorithm yielded the lowest (6.66 ± 3.48 mm) among field-based estimation algorithms. The projection algorithm required fewer stimuli, possibly resulting from its suitability for the grid-based mapping data collection method. The TMS cortical hotspots from all algorithms consistently deviated from the fMRI activation peak (20.52 ± 8.46 mm for five algorithms).

**Conclusion:**

The association algorithm might be a superior choice for clinical applications and basic neuroscience research, due to its lower prediction error and higher estimation sensitivity in the deep cortical structure, especially for the sulcus. It also has potential applicability in various other TMS domains, including language area mapping and more. Otherwise, our results provide further evidence that TMS motor mapping intrinsically differs from fMRI motor mapping.

## 1 Introduction

Transcranial magnetic stimulation (TMS) is a non-invasive focal brain stimulation technique widely used in brain mapping studies (Ilmoniemi et al., 1999; Siebner et al., 2009; Lefaucheur, 2019). When a single supra-threshold TMS pulse is applied to the motor cortex, a motor-evoked potential (MEP) may be recorded from the targeted muscle, such as the first dorsal interosseous muscle (FDI). TMS motor mapping, in which multiple MEPs typically recorded from predetermined stimulation sites on a grid are used to non-invasively probe motor cortex representation, is one of the most important applications of TMS (Wilson et al., 1993; Sondergaard et al., 2021). TMS has several advantages over other noninvasive approaches to motor cortex mapping such as functional magnetic resonance imaging (fMRI). Compared to fMRI, TMS motor mapping is in closer agreement with direct cortical stimulation (DCS) mapping, which is regarded as the current gold standard for delineating the motor cortex (Krieg et al., 2012; Coburger et al., 2013; Mangraviti et al., 2013). Moreover, TMS requires less patient cooperation such as performing motor tasks, which is difficult for patients with paresis or plegia or children with autism or developmental delay (Narayana et al., 2015, 2021; Braden et al., 2022). Such advantages have made TMS motor cortex mapping promising in clinical applications, such as pre-surgical planning (Takahashi et al., 2013; Lefaucheur and Picht, 2016), risk stratification (Rosenstock et al., 2017), motor rehabilitation (Lüdemann-Podubecká and Nowak, 2016) and basic research such as developmental plasticity (Narayana et al., 2015; Grab et al., 2018; Babwani et al., 2021).

Given a set of recorded MEPs as well as the corresponding stimulating sites on the scalp, there are various algorithms, with increasing complexity, for the prediction of the location and spread of the motor cortex. The most traditional and simplest one is called the projection algorithm, which assumes that the effect of a TMS pulse at a scalp site can be reduced to a single point projected onto the cortex (Ruohonen and Karhu, 2010; Julkunen, 2014; Kraus and Gharabaghi, 2015). Simple geometric models cannot characterize the effect of TMS on the cortex well. Therefore, several approaches have been introduced that numerically simulate the electric field induced by TMS, taking into account the coil orientation and the complex geometry of the individual brain (Thielscher et al., 2011; Laakso et al., 2014; Reijonen et al., 2020). Analogous to the projection algorithm, the projection point was substituted by the peak point of the induced electric field on the cortex (called max-EF algorithm here) (Ruohonen and Karhu, 2010; Sollmann et al., 2016; Novikov et al., 2018). But it’s still geared to point-based algorithms, rather than field-based algorithms that utilize complete information from the electric field distribution. Opitz et al. hypothesized that when a recorded MEP was large, the induced electric field should be concentrated near the target region and vice versa. Based on this assumption, they used each MEP to weight the corresponding electric field and used the weighted average electric field to estimate the motor cortex (called EF-COG algorithm here) (Opitz et al., 2013). Other studies pointed out that, in the targeted motor cortex, there should be a strong association between the MEP and the corresponding electric field strength. Thus, they evaluated the degree of association in each cortical patch to estimate the motor cortex (called the association algorithm here) (Thielscher and Kammer, 2002; Matthäus et al., 2008; Laakso et al., 2018; Weise et al., 2020; Kataja et al., 2021; Numssen et al., 2021; Weise et al., 2023). Moreover, some algorithms borrowed from the idea of electroencephalography source localization and performed a minimum norm estimation (called MNE algorithm here) to estimate the extent of the motor cortex (Bohning et al., 2001; Pitkänen et al., 2017; Reijonen et al., 2022).

With the emergence of new estimation algorithms for motor mapping, the comparison of different approaches is becoming a growing concern. For example, Seynaeve et al. compared the motor map from the projection, max-EF, and EF-COG algorithm with the DCS mapping result as a standard (Seynaeve et al., 2019). However, it is difficult in practice to obtain DCS data, and the mapping accuracy of DCS mapping is limited by finite discrete sampling (Seynaeve et al., 2019). Fortunately, it has been found that the electric field modeled numerically in the target brain area is a great predictor of the neurophysiological or behavioral response induced by transcranial brain stimulation (Argyelan et al., 2019; Jamil et al., 2020; Fridgeirsson et al., 2021; Mosayebi-Samani et al., 2021). Several studies have been concerned with the potential of optimizing coil position according to the electric field simulation (Weise et al., 2020; Gomez et al., 2021), and Reijonen et al. took the difference between electric-field-based optimized coil position and measured scalp hotspot coil position as the performance index for the MNE algorithm based on realistic and spherical head models (Reijonen et al., 2022). This suggests that the distance between the optimized coil position and measured scalp hotspot coil positions could serve as a viable and practical performance metric for comparing different estimation algorithms.

The number of data points (stimuli) fed into an estimating algorithm is closely related to the stability of the motor map and the acquisition time of mapping data. There is a trade-off between motor map stability and acquisition time. The more stimuli, the greater the stability, but the longer the acquisition time, which leads to practical difficulties (Sinitsyn et al., 2019; Sollmann et al., 2021; Sondergaard et al., 2021). Thus, the minimum number of stimuli required to deliver a stable mapping result is another valuable performance index in the comparison of various estimation algorithms. Pitkänen et al. inferred that the MNE algorithm might need fewer stimuli because of the higher resolution capacity of its mapping, compared with the projection algorithm (Pitkänen et al., 2017). However, no study has investigated the number of stimuli required for currently available algorithms simultaneously, and thus there is no evidence showing which algorithm requires the least number of stimuli.

The results of previous studies have suggested that the cortical hotspot location from TMS mapping based on the projection algorithm was inconsistent with the peak location of fMRI motor task activation, and the TMS cortical hotspot was always located more anterior (Herwig et al., 2002; Lotze et al., 2003; Diekhoff et al., 2011). This has been ascribed to neurophysiological differences, i.e., neurons activated by TMS and those detected by fMRI differed (Herwig et al., 2002; Wang et al., 2020). However, advanced field-based estimation algorithms have the potential to improve the estimation performance of motor mapping (Seynaeve et al., 2019). Thus, it is important to revisit the incongruency in cortical hotspot locations estimated by TMS and fMRI for advanced algorithms.

Given the above, this study aims to simultaneously compare the aforementioned five estimation algorithms on one set of TMS mapping data. We mainly conducted two experiments to compare them: first, we compared the distance between the measured scalp hotspot and optimized coil position according to the mapping results from all algorithms; second, we probed the relationship between the number of stimuli and estimation stability to determine the minimum number of stimuli required to deliver a stable mapping result for each algorithm. We also investigated whether inconsistencies between TMS and fMRI cortical hotspot locations still exist when considering the induced electric-field distribution in the estimation process.

## 2 Method

### 2.1 TMS data acquisition

TMS mapping data were obtained from our previous study (Jiang et al., 2020). Twelve healthy right-handed participants (7 males, 22 ± 2.7 yr) were recruited. None of them had any contraindications to TMS or any history of neurological or psychiatric diseases. All participants provided written informed consent before the experiment. The protocol was approved by the ethics committee of the State Key Laboratory of Cognitive Neuroscience and Learning at Beijing Normal University. TMS motor mapping was conducted using a Magstim rapid2 (Magstim Ltd., Dyfed, United Kingdom) with a D70 Air Film figure-of-eight coil. We designed a 6 × 7 stimulation grid that covered the motor-related area in the left hemisphere, according to the motor-related functional transcranial brain atlas (Jiang et al., 2020). The grid spacing was 3 continuous proportional coordinate (CPC) units, which are normalized scalp coordinates with inter-individual comparability (Xiao et al., 2018), and the group average Euclidean distance of a unit was around 1 cm (see Supplementary Figure S1A). 1 cm^2^ stimulation grid is widely adopted (57/75 studies) (Sondergaard et al., 2021), making the comparison results suitable for the majority of scenarios of motor mapping. The coil was placed tangentially to the scalp with the coil handle pointing backward and laterally at 45° away from the midline, which is the optimal orientation to induce MEP (Balslev et al., 2007; Reijonen et al., 2020). The resting motor threshold (RMT) was defined as the lowest intensity eliciting a minimum peak-to-peak amplitude of 50 μV in at least 5 of 10 TMS pulses (Rossini et al., 2015). The stimulation intensity for mapping was set to 120% RMT, resulting in more reliable MEP responses (Ngomo et al., 2012). The best coil position for evoking the largest MEPs in the first dorsal interosseous (FDI) muscle, the resting motor threshold (RMT) was found and recorded.

For reliable measurement of MEP, we delivered 6 TMS pulses per site in the grid with interstimulus intervals of over 5 s (Cavaleri et al., 2017; Nazarova and Asmolova, 2021; Sondergaard et al., 2021). During stimulation, the subjects were asked to maintain complete muscle relaxation. Peak-to-peak amplitudes were recorded from the subjects’ FDI muscle in the right upper limb with bipolar surface electrodes using a Brainsight EMG Isolation Unit and Amplifier Pod (Rogue Research Inc., Canada). The measurement of the RMT and input–output (I/O) curve demonstrated that the FDI muscle was more reliable than the abuctor pollicis brevies muscle (Malcolm et al., 2006), both of which are commonly used muscles in TMS motor measurement.

### 2.2 Estimation algorithms for motor mapping

Head modeling and electric field simulation were realized in the SimNIBS v3.2 open-source pipeline (Thielscher et al., 2015) (supplement). The recorded MEPs and stimulation positions (or electric fields) were used to estimate the motor cortex via each algorithm. Since the entire cortical surface consisted of over two hundred thousand triangles leading to a large amount of useless computation, before estimation, an estimation scope was determined by projecting the stimulation grid onto the cortical surface and expanding it by 0.5 cm (see Supplementary Figure S1B).

**Figure 1:**
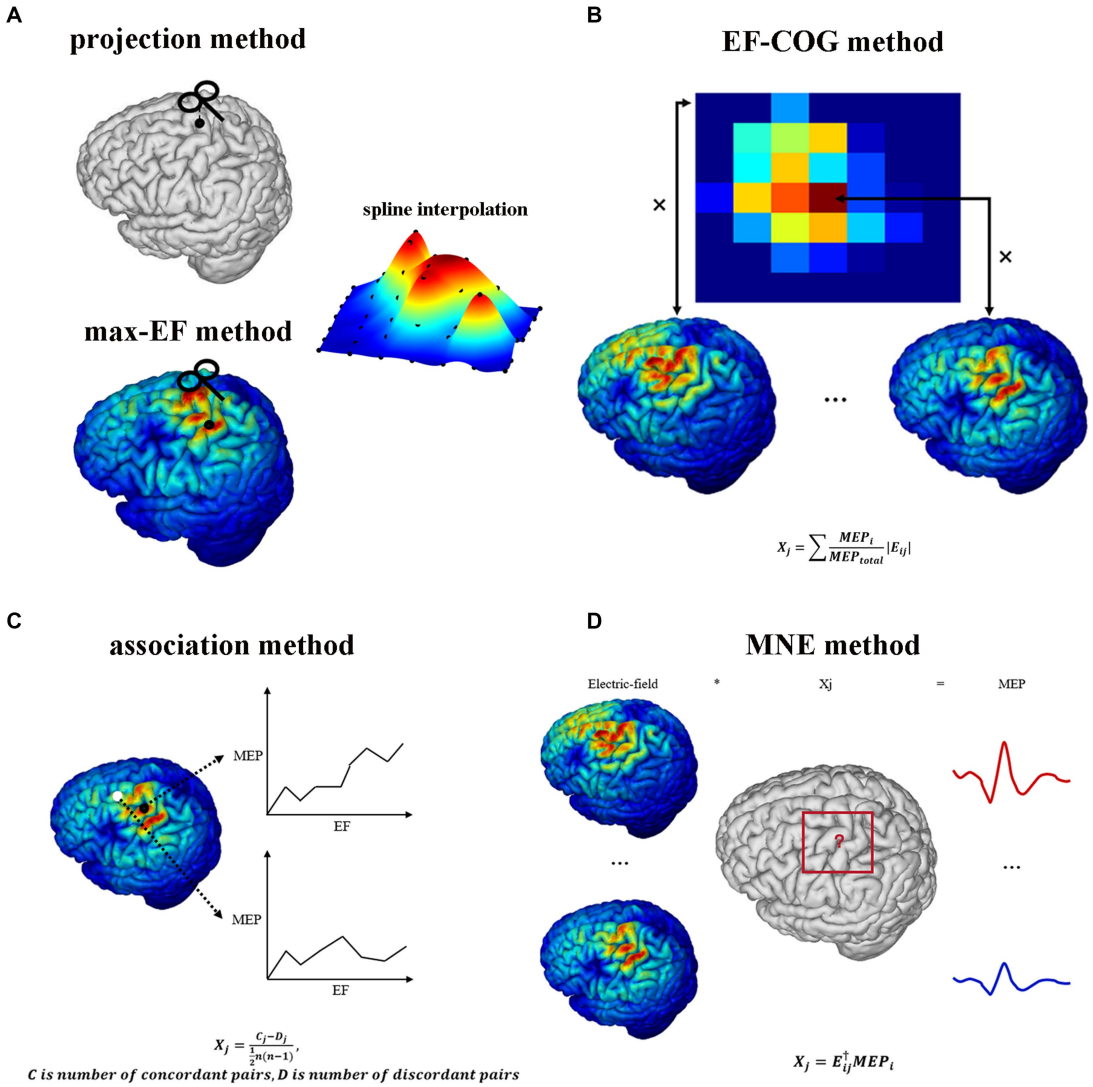
TMS motor cortex estimation scheme of five algorithms. It shows each algorithm’s logic and mathematical description of part algorithms. **(A)** Projection algorithm and max-EF algorithm. **(B)** EF-COG algorithm. **(C)** Association algorithm. **(D)** MNE algorithm. In the mathematical description, Xj represents the probability that the jth cortical patch belongs to the motor cortex; E_ij_ represents the electric field strength of the jth cortical patch in the ith stimulation; MEP_j_ represents the MEP value recorded in the ith stimulation; MEP_total_ represents the sum of all recorded MEP.

Figure 1 shows the estimating schemes of five algorithms. Two point-based algorithms initially identify the cortical sites most likely to be influenced at each point within the stimulation grid. Then they undertake the interpolation on the cortical surface using MEP values corresponding to each cortical site, thereby generating a continuous estimated motor map. In the projection algorithm (Figure 1A), the cortical site most likely to be influenced is determined using the Möller–Trumbore intersection algorithm, which identifies the cortical site nearest to the normal of the TMS coil surface (Möller and Trumbore, 1997). In the max-EF algorithm, the cortical site is identified as the location with the highest electric field strength at the 99.9th percentile. The selection of the 99.9th rather than 100th is intended to mitigate the boundary effects of the electric field (Saturnino et al., 2019). To enable interpolation on the 3D cortical surface (Julkunen, 2014), we initiated the process by mapping the pre-identified cortical sites onto the 2D plane parallel to the gyrus (van de Ruit et al., 2015; Jonker et al., 2019). Subsequently, we conducted interpolation of the MEP values through the implementation of a cubic spline algorithm. The interpolated values were then projected from the 2D plane to the 3D cortical surface using the Nearest-neighbor interpolation algorithm.

Opitz et al. referenced the TMS COG position from traditional TMS motor mapping, which calculates a MEP “Center of Gravity,” signifying a scalp position where a large MEP is reliably produced (Sondergaard et al., 2021). They introduced the concept of the electric field “Center of Gravity” (Opitz et al., 2013), portrayed as a probability map of the motor cortex. In the EF-COG algorithm (Figure 1B), this concept is realized by conducting a weighted sum of the electric field strength associated with MEPs. The fundamental concept underlying the association algorithm is predicated on the identification of the motor area as the cortical region characterized by a robust correlation between the surrounding electric field strength and the corresponding MEP values. We calculated the Kendall’s rank coefficient between the electric field strength and MEPs referred to as Matthäus et al. (2008). The resultant coefficient serves as a representation of the estimated motor map (Figure 1C). The MNE algorithm is rooted in source localization methodologies commonly employed in electroencephalography (Bohning et al., 2001; Pitkänen et al., 2017; Reijonen et al., 2022). It established a computational model to delineate how MEPs are determined by the distributions of electric field strength under each stimulation. In this model, the distribution of electric field strength is the independent variable, the MEP value is the dependent variable, and the unknowns represent the probability of a cortical patch belonging to the motor area. This model is undetermined due to having fewer dependent variables than unknowns. To address this, Wiener regularization is applied to resolve the problem, resulting in an estimated motor map (Pitkänen et al., 2017).

### 2.3 Similarity of estimation results

The similarities and differences among mapping results from all five estimation algorithms were investigated in several spatial scales: the entire estimated motor map, map maxima (cortical hotspot), and center-of-gravity (COG). The Pearson correlation coefficient (r) was computed as the map level similarity between each pair of algorithms’ maps. The Euclidean distance between each pair of cortical hotspots was computed as the cortical hotspot similarity index. The Euclidean distance between each pair of COGs was computed as the COG similarity index. The non-parametric Wilcoxon signed-rank test was used to check that there exists a statistically significant difference between pairs of cortical hotspots or COGs. Account for the folded structure of the cortex, we also adopted the geodesic distance to measure the difference of cortical hotspot location estimated by five algorithms. The geodesic distance of two cortical hotspots was calculated with tvb-gdist 2.1.0.

### 2.4 Distance between measured scalp hotspot and optimized coil position

**Figure 2:**
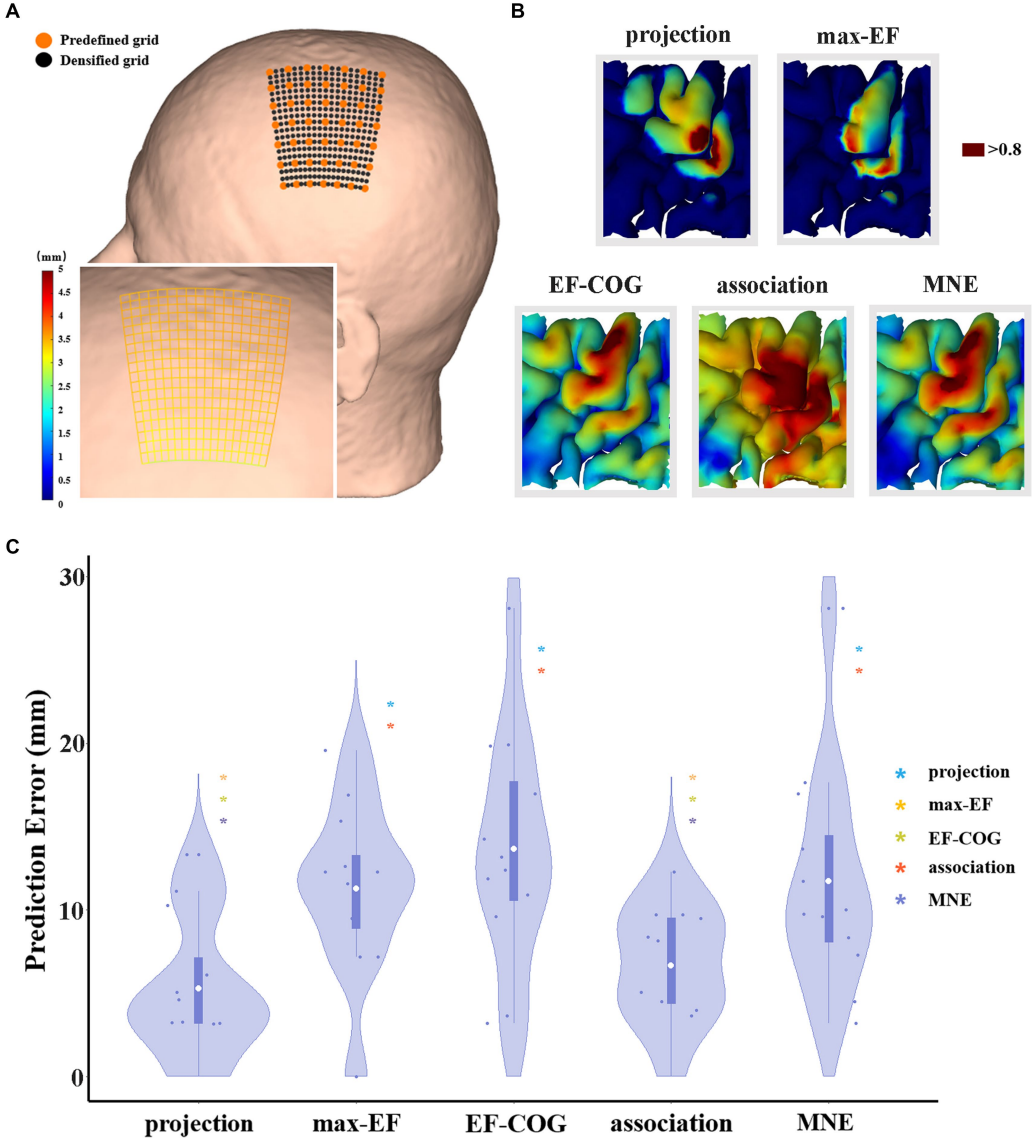
The comparison of the prediction error of five algorithms. **(A)** It shows a densified grid exampled on subject 2. The black dot represents the stimulation grid predefined before the experiment, and the orange dot represents the added grid points in the simulation. A zoom-in sub-graph in the left-bottom shows the block distance between any two grid points represented by the gradient color; **(B)** Exampled as subject 4, it shows the distribution of hand area estimated by five algorithms, and the dark red area represents the remaining hand location at the threshold of 0.8; **(C)** Violin plots show the prediction error of five algorithms at the threshold of 0.8. For each algorithm, the prediction error of each subject is represented by the blue dot. The white dot represents the group-average prediction error. Asterisks indicate a significant difference between the prediction error of the algorithm plotted and that of another algorithm (represented by different colors). *p < 0.05, **p < 0.01, ***p < 0.001.

The scalp hotspot is the scalp position where TMS induces maximum MEP response during the motor mapping experiment. The optimized coil position within a mapping algorithm is delineated as the theoretical scalp position capable of inducing the maximum MEP response corresponding to the motor cortex, as estimated by the algorithm. To assess the estimation accuracy of each algorithm, we need to calculate the distance between the optimized coil position and the scalp hotspot position. The shorter distance might mean a more accurate estimation algorithm. The distance is regarded as the prediction error here. Before optimization, we densified the predefined grid to shorten the grid spacing (Figure 2). We fixed the stimulation orientation in the experiment, so we did not consider the influence of orientation when optimization. Then we did an electric field simulation on each densified grid point, and determined the optimized coil position by the maximum electric field strength in the estimated motor cortex. The prediction error data was non-parametric (Shapiro–Wilk normality test), thus differences between algorithms were tested using Kruskal–Wallis’s test for independent data. All data met the sphericity assumption, assessed with Mauchly’s test. A false-discovery-rate correction was used for multiple comparisons. For all statistical analyses, a *p*-value of <0.05 was considered significant.

The threshold for outlining the motor cortex is crucial when optimization. However, it is unclear and there is no consensus on how to select the outlining threshold, and whether a uniform threshold should be selected for all algorithms. Thus, we normalized the estimation value to reasonably set the same outlining threshold, and explore the difference in estimation accuracy under various thresholds (0.5–0.9). We selected 0.8 as the recommended threshold because the group average area of the motor cortex estimated by the projection algorithm is close to 270 mm^2^ proposed by previous studies (Pitkänen et al., 2017; Nazarova et al., 2021) (see Supplementary Figure S3).

### 2.5 Relationship between the number of stimuli and estimation stability

To identify the minimum number of stimuli (N_min_) needed for stable mapping results, we investigated the relationship between a number of stimuli (MEP from 1 stimulus = average MEPs from 6 pulses) and stability for each algorithm. We subsampled the original stimulation data (mean MEPs >50 μV, as standard in TMS) to estimate the motor map for a smaller number of stimuli. For each given number of stimuli, the subsampling process was randomly conducted 1,000 times (Efron, 1979). Stability was defined as the average Pearson correlation coefficient between the 1,000 maps obtained from subsampling (sub-sample map) and the map obtained from the original data (original map).

The N_min_ for each algorithm was defined as the minimum number of stimuli needed to reach a highly stable level when the Person correlation coefficient between maps from sub-sample data and the original data reached 0.9. We conducted statistical analysis in the same method as the comparison of prediction error. In addition, considering correlation analysis might be biased in favor of algorithms that yield a more diffuse map (e.g., the EF-COG algorithm), we also calculated the N_min_ at which the distance between the peak region (top 5% within the search scope) in the sub-sample maps and the original map is reduced to less than 3 mm.

### 2.6 Comparing the motor mapping of TMS and fMRI

Each subject’s fMRI data, based on gradient-echo echo planar imaging (EPI) sequences were also acquired on 332 Siemens Trio 3 T MRI Scanner (32 axial slices; repetition time (TR) = 2000; echo time (TE) = 28 ms; flip angle (FA) = 90°; field of view (FOV) = 102 × 102 mm; 51 × 51 matrix size with a resolution of 2 × 2 mm^2^) during a finger tapping task. To mitigate the differences between TMS and fMRI mapping arising from the movement of different muscles, volunteers performed right index finger tapping to activate the FDI muscle at a fixed frequency. In studies comparing the fMRI and TMS, a hand movement task lasting 20–40 s, alternating with rest, was commonly employed, with the majority using 6 blocks (3/5 studies, see Supplementary Table S1). In our study, the task consisted of seven rest blocks of 24 s each, featuring a fixation point, alternating with six task blocks of 24 s each. To ensure the stability of the volunteers, we added a rest block at the beginning of the task.

To acquire images with a higher spatial and temporal resolution, the above fMRI scanning only covered the upper part of the cerebrum containing the motor cortex, from the anterior and posterior commissure to the vertex, so an additional whole EPI volume was acquired for co-registration (96 axial slices; TR/TE/FA = 6000/28 ms/90°; FOV = 102 × 102 mm; 51 × 51 matrix size with a resolution of 2 × 2 mm^2^). The analysis of fMRI data is described in the supplement.

The identification of the TMS cortical hotspot has traditionally been defined based on the projection algorithm and can be generalized to other algorithms to find the cortical location with the map maxima. The fMRI activation peak was determined as the point with the highest z-statistic in the estimation scope. The cortical sites were transformed into the Montreal Neurological Institute (MNI) space using the non-linear deformation field, which was obtained by segmenting and spatially normalizing the T1 image using Statistical Parametric Mapping 12. Then, we calculated the Euclidean distance between each algorithm cortical hotspot and the fMRI peak for each subject. We further calculated the divergence in X, Y, and Z coordinates (in the MNI coordinate system) to investigate the directional bias of the TMS cortical hotspot. Similarly, we also investigated the COGs to examine the spatial mismatch between TMS mapping and fMRI activation. The non-parametric Wilcoxon signed-rank test was used to check if there exists a statistically significant difference between each algorithm’s results and fMRI activation results.

## 3 Results

### 3.1 Estimated motor maps

**Figure 3:**
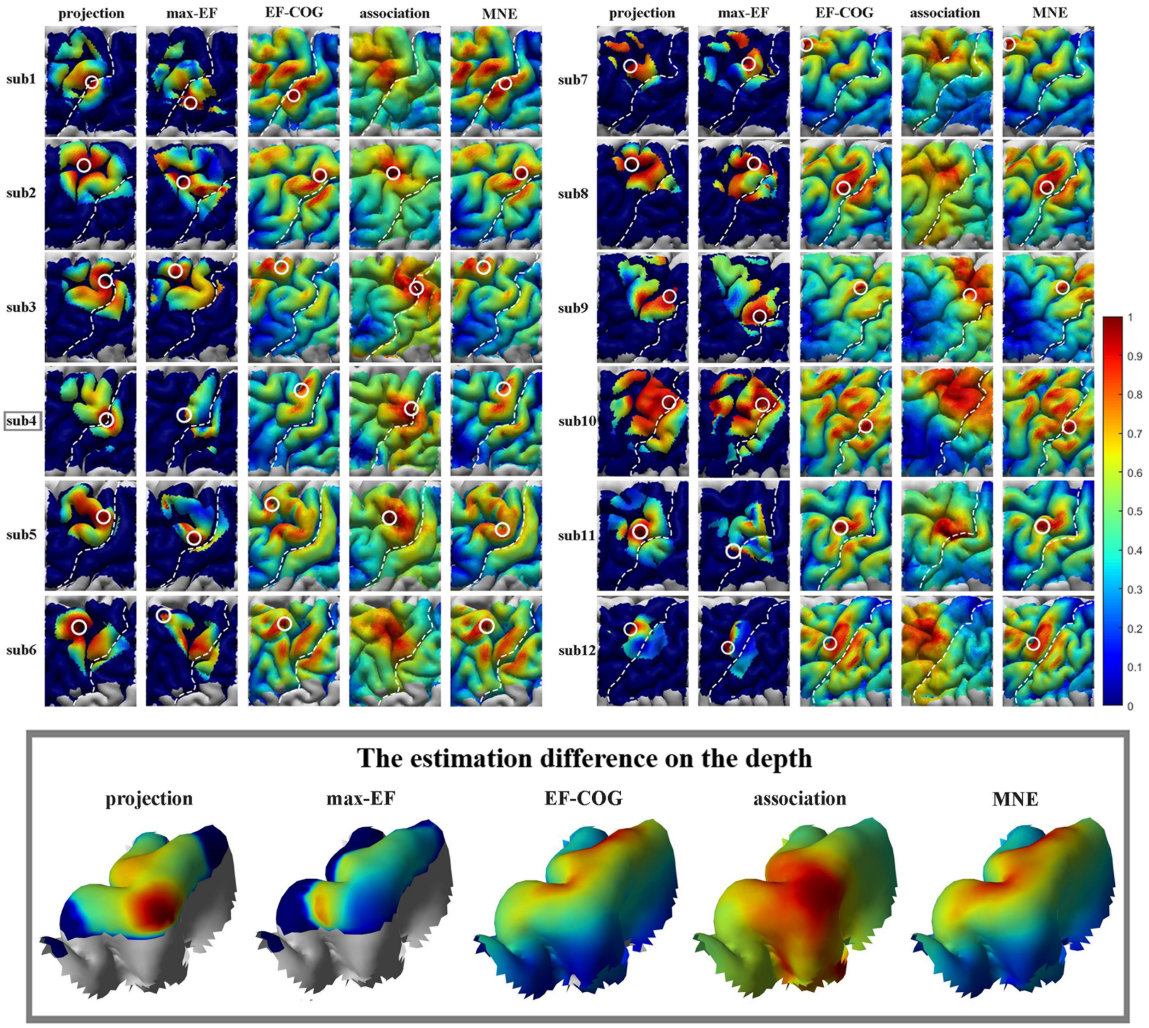
Estimated motor maps from five algorithms. The left panel shows motor maps of subjects 1–6, and the right panel shows subjects 7–12. The bottom panel displays the estimation results of five algorithms around the omega region, as illustrated by subject 4. White circles mark the cortical hotspots, and white dotted lines mark the central sulcus. The color bar represents the normalized estimation value, with red indicating a higher probability of inclusion in the motor cortex and blue indicating a lower probability.

**Figure 4:**
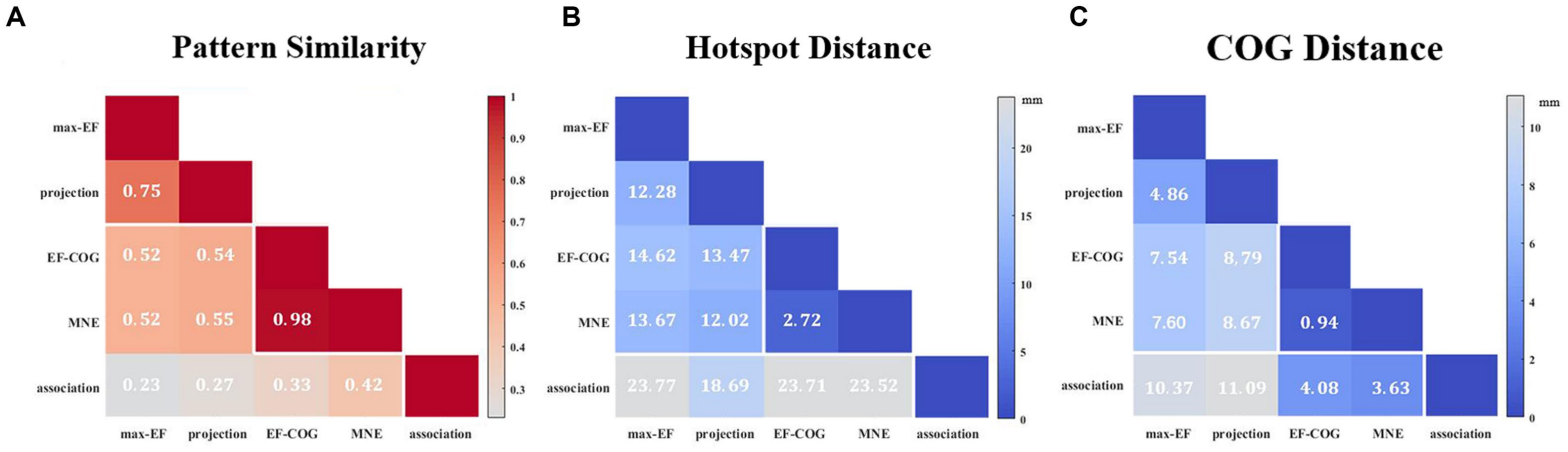
Similarity of motor maps from different algorithms. The similarity in terms of **(A)** pattern similarity of the motor map in terms of correlation coefficient r, **(B)** the Euclidean distance between cortical hotspots, and **(C)** the Euclidean distance between COGs. White numbers and shading color indicate the group’s average value.

The results from the five algorithms were normalized and shown for each subject (*N* = 12) in Figure 3. The projection and max-EF algorithms yielded more concentrated motor maps than the others. Interestingly, the estimation sensitivity varies among the five algorithms in the sulcus. Interpolating solely on a 2D plane, the point-based algorithms are incapable of estimating values in the sulcus. Of the field-based algorithms, the association algorithm identified half of the subjects’ estimated cortical hotspots in deep structures, while the others were located on the gyrus. In the bottom panel of Figure 3, the estimation results around the omega region (e.g., subject 4) are presented, which serves as the anatomical landmark for the hand area (Yousry et al., 1997). To facilitate a comparison of the estimation results from the five algorithms, we filled in the 0 values in the sulcus of the point-based algorithm’s estimation results (Figure 3, top panel). We quantitatively investigated the pattern similarity between the estimated maps and the distance between cortical hotspots or COGs (Figure 4) from the five algorithms. Two pairs of algorithms yielded maps with a strong similarity: EF-COG and MNE (r = 0.98 ± 0.02, mean ± SD); projection and max-EF (r = 0.75 ± 0.09). The grid spacing of the predefined grid is 1CPC (3.36 ± 0.14 mm, Figure 4A). The pairwise correlation was statistically significant in 12 subjects (*p* < 0.001). The cortical hotspot did not significantly differ between EF-COG and MNE algorithms (*p* = 0.125), and the distance was 2.74 ± 6.65 mm (Figure 4B). It showed homogenous results when substituting cortical hotspots’ geodesic distance (see Supplementary Figure S2). The remaining pairwise cortical hotspots differed significantly (*p* < 0.001) with the mean distance all over 12 mm. The shortest distance of COGs was between EF-COG and MNE (0.94 ± 0.74 mm, *p* < 0.001) (Figure 4C).

### 3.2 Comparison of prediction error

Prediction error was significantly different among the five algorithms (df = 4, *F* = 7.269, *p* < 0.001), and Figure 2C shows pair-wise comparison results. At the threshold of 0.8, projection and association algorithms have significantly lower prediction errors than the other three algorithms (projection = 5.27 ± 4.24 mm, association = 6.66 ± 3.48 mm, max-EF = 11.28 ± 5.09 mm, EF-COG = 13.66 ± 6.98 mm, MNE = 11.73 ± 6.75 mm), and the two of them have no significant difference (*p* = 0.386). Supplementary Figure S4 shows the monotonously decreasing prediction error for the projection and association algorithms, but monotonously increasing for the other three algorithms with the increasing of the cutting threshold. In the range of 0.75 to 0.9, the projection and association algorithms keep a significantly lower prediction error than others.

### 3.3 Comparison of the minimum number of required stimuli

**Figure 5:**
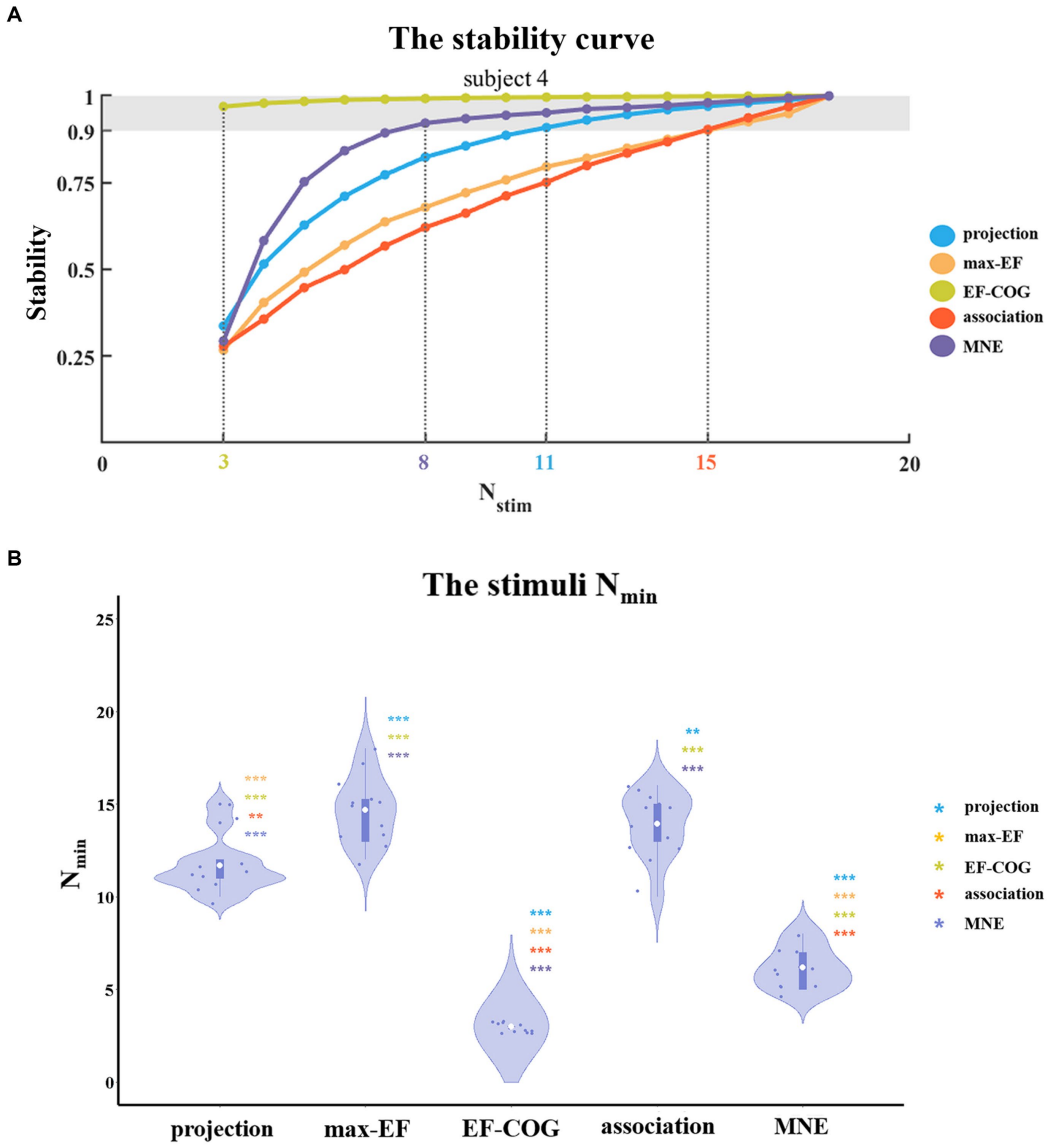
The comparison of a minimum number of required stimuli. **(A)** It shows the relationship between the number of stimuli and the stability of five algorithms. Examples are given for subject 4. Color numbers show the N_min_ of the five algorithms, the number of stimuli required for stability > = 0.9 (shade region); and **(B)** Violin plots show the distribution of the N_min_ of the five algorithms. For each algorithm, the N_min_ of each subject is represented by the blue dot. The white dot represents the group-average N_min_ of each algorithm. Asterisks indicate significant differences between the N_min_ of the algorithm plotted and that of another algorithm (represented by different colors). *p < 0.05, **p < 0.01, ***p < 0.001.

To determine the N_min_ required to produce a stable map, we probed the relationship between the number of stimuli and the estimation stability of each algorithm. Figure 5A shows example curves from one typical subject (subject 4). With the increase in the number of stimuli, the stability of all five algorithms increased monotonously. The ranking of N_min_ of the five algorithms was: EF-COG < MNE < projection < max-EF = association. EF-COG algorithm required only 3 stimuli to estimate stably (stability = 0.969 ± 0.327), and max-EF and association required 15 stimuli (max-EF stability = 0.901 ± 0.055, association stability = 0.903 ± 0.061). N_min_ was significantly different among the five algorithms (df = 4, *F* = 187.362, *p* < 0.001), and Figure 5B shows pair-wise comparison results. Group-level analysis revealed that the EF-COG algorithm required the least N_min_ (3 ± 0), which was significantly less than each of the other four algorithms (*p* < 0.001). Max-EF and association algorithm required the most N_min_ (max-EF 14.75 ± 1.76; association 14.00 ± 1.81), and no significant difference was found between them (*p* = 0.685). The group average and SD of N_min_ were 6.17 ± 1.11 for MNE, and 11.67 ± 1.50 for projection, which both were significantly different from the other four algorithms. In the investigation of the N_min_ for stable peak region, the EF-COG algorithm still had the smallest N_min_ (see Supplementary Figure S5).

### 3.4 Comparison of TMS and fMRI motor mapping

**Figure 6:**
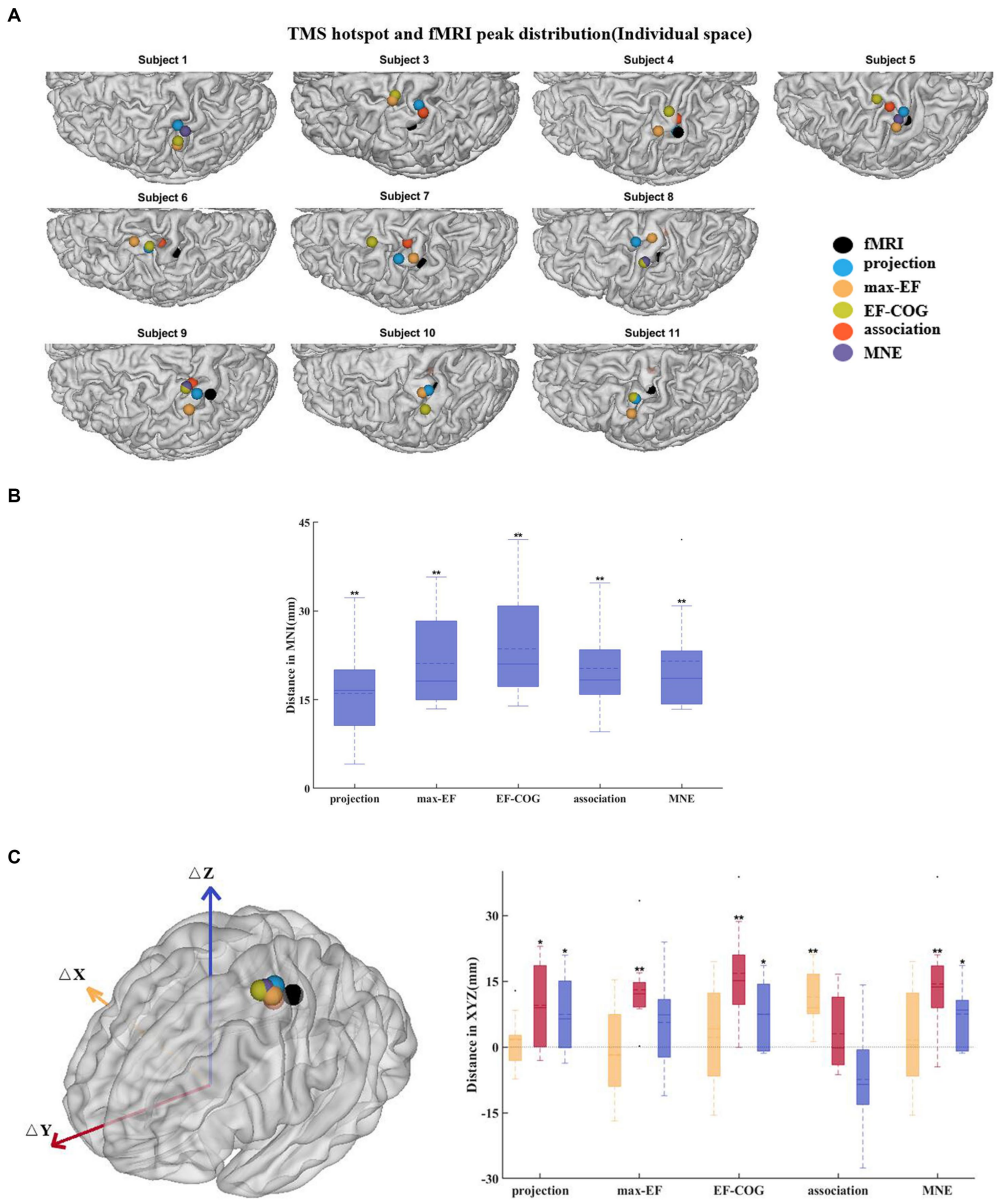
Divergence between TMS cortical hotspots and fMRI peaks. **(A)** Cortical hotspots were estimated by five algorithms and fMRI peaks (black spheres) in individual MRI spaces. **(B)** Euclidean distance between the cortical hotspots and fMRI activation peaks. **(C)** The left panel shows group-mean cortical hotspots and fMRI peaks. The right panel shows a divergence between TMS cortical hotspots and fMRI peaks separately in three axes (red, Y-axis; blue, Z-axis; yellow, X-axis). All box plots show median (black solid line), mean (black dashed line), interquartile range (box top and bottom), and 10th and 90th percentiles (error bars). *p < 0.05, **p < 0.01.

Subjects 2 and 12 were not included because they had no significant fMRI activation. The remaining subjects’ activation peaks were all located in the central sulcus, but most of the TMS cortical hotspots were located in the precentral gyrus (Figure 6A). The fMRI peak site significantly differed from all cortical hotspot sites estimated by the five algorithms (distance_projection_ = 16.07 ± 8.41 mm, distance_max-EF_ = 21.13 ± 7.70 mm, distance_EF-COG_ = 23.59 ± 9.17 mm, distance_association_ = 20.28 ± 7.77 mm, distance_MNE_ = 21.52 ± 9.00 mm, *p* = 0.002) (Figure 6B). In the Y-axis direction, TMS cortical hotspots were located significantly more anterior to the fMRI peak for projection, max-EF, EF-COG, and MNE algorithms (projection *p* = 0.02; max-EF *p* = 0.002; EF-COG *p* = 0.004; MNE *p* = 0.004), but not significantly for association algorithm (*p* = 0.492) (Figure 6C). In the Z-axis direction, TMS cortical hotspots were located significantly more superior to the fMRI peak for projection, EF-COG, and MNE algorithms (*p* = 0.027). In the X-axis direction, no statistically significant differences between the TMS cortical hotspot and fMRI peak were found. Similar results were found for COG (see Supplementary Figure S6).

## 4 Discussion

### 4.1 The estimated motor maps of five algorithms

Based on our results (Figure 3), the distribution was more centralized for the projection and max-EF algorithms, which is consistent with previous studies (Pitkänen et al., 2017; Seynaeve et al., 2019). One possible explanation for this is that the projection and max-EF algorithms work based on points and do not consider the spread of neuronal activity induced by TMS, while the other three field-based algorithms work based on the electric field distributions. Notably, approximately half of the subjects’ cortical hotspots estimated from the association algorithm were located in the deeper cortex. It is possibly attributed to the association algorithm’s higher sensitivity to electric field strength compared to the MNE and EF-COG algorithms. The point-based algorithm is unable to depict the probability distribution of the motor cortex in the sulcus due to the lack of a reliable and physiologically valid interpolation method for the 3D cortex. We adopted a common and demonstrated repeatable 2-D spline interpolation method (Wilson et al., 1993; Borghetti et al., 2008; Julkunen, 2014; Jonker et al., 2019). Although the MNE algorithm had a much higher computational complexity, its results were highly similar to those of the EF-COG algorithm (Figure 4). The reason for this is not clear, but it may be due to the application of Wiener regularization to reduce the effect of MEP variability (Numminen et al., 1995; Pitkänen et al., 2017). Therefore, besides improving the accuracy of the hypothesized forward model, the performance of the MNE algorithm may also be enhanced by selecting an appropriate regularization method.

### 4.2 Comparison of estimation effectiveness and efficiency among five algorithms

We compared the effectiveness and efficiency of five different estimation algorithms mainly through two experiments. In the first experiment, we evaluated the prediction error of scalp hotspots for each algorithm as a measure of its estimation effectiveness. The projection and association algorithms produced the lowest prediction (5.27 mm, 6.66 mm) in the point-based and field-based estimation algorithms, respectively. Under the lower outlining threshold, such as 0.5, the prediction error might depend on the search scope range restricted before estimation. It could lead to similar evaluations for all algorithms because the remaining hand area occupies over half of the search scope. With an increasing threshold, the prediction error becomes more dependent on the estimation accuracy rather than the search scope range. Our results indicate that the projection and association algorithms consistently performed better than other algorithms in terms of lower prediction errors over the threshold range of 0.6 to 0.9, with a statistically significant difference observed in the range of 0.75 to 0.9 (see Supplementary Figure S4). The area in the chosen range was regarded as the hotspot extent, which outlines the area where the highest MEPs occur (Reijonen et al., 2020). Considering that prediction error is influenced by errors in scalp hotspot measurement and the selection of the electric field component (Bungert et al., 2017), we have provided two supplements. Firstly, we have interpolated measured MEPs on the densified grid and determined the maximum site to replace the measured scalp hotspot position. This resulted in the projection and association algorithms still performing the best (*p* < 0.01 in the pair-wise comparison). Secondly, when optimizing the coil position, we substituted the electric field strength with components of the field that are normal and tangent to the local cortex orientation, respectively. Both algorithms produced the lowest prediction error when using the tangent component (*p* < 0.01) and produced a significantly lower prediction error than the EF-COG algorithm when using the normal component (*p* < 0.05). These results reliably suggest that the projection and association algorithms are more effective.

Several studies have shown that both the projection and EF-COG algorithms perform well in motor mapping when taking DCS mapping results as the standard (Coburger et al., 2011, 2013; Opitz et al., 2014; Seynaeve et al., 2019). Seynaeve et al. suggested that the projection and EF-COG algorithms both estimate the motor cortex with high accuracy (85 and 78% respectively) and that the EF-COG algorithm is better at capturing the entire motor cortex representation than the projection algorithm (Seynaeve et al., 2019). In our study, the projection and association algorithms demonstrated lower prediction errors than others. The association algorithm can outline the entire distribution of the motor cortex without neglecting its deep structures, such as the gyrus lip and sulcus (Figure 3).

Although the projection algorithm is unable to estimate in the sulcus, it still had a similarly better prediction error compared to the association algorithm. This might be related to modeling research suggesting that the primary target of TMS is the crown top and lip regions of cortical gyri (Bungert et al., 2017; Siebner et al., 2022), which can be estimated by the projection algorithm. The part findings of our research are supported by Seynaeve et al., who suggested that the projection algorithm exhibited higher estimation accuracy than the EF-COG algorithm standardized as DCS mapping (Seynaeve et al., 2019). The prediction error of the MNE algorithm in our research is 11.73 mm, whereas it was 7.0 mm in the previous research that used the same method to evaluate the effectiveness of the MNE algorithm (Reijonen et al., 2022). The lower prediction error observed in our research may be attributed to the absence of the I/O curve in the MNE algorithm replication.

We aimed to explore the minimum number of stimuli required for stable mapping with each algorithm in the second experiment of the simulation. In addition to providing a complete depiction of the distribution of the motor cortex in three-dimensional space, the collection of multiple TMS stimuli is utilized to mitigate the effects of MEP variability (Cavaleri et al., 2017; Sinitsyn et al., 2019). In this study, we employed the classic method of collecting TMS data, which involves an even stimulation grid (Sondergaard et al., 2021). It means that the number of stimuli refers to the number of stimulation grid points that contain six TMS pulses. It mitigates the effects of MEP variability by repeatedly sampling MEPs at the same site and obtaining a more stable MEP measurement (Cavaleri et al., 2017; Therrien-Blanchet et al., 2022). With the development of neuronavigation and electric field modeling, several studies have proposed that the collection of a single TMS pulse can be directly used for motor mapping (van de Ruit et al., 2015; Numssen et al., 2021; Sondergaard et al., 2021). It mitigates the effects of MEP variability by capturing more spatial information and obtaining a more stable distribution of the motor cortex. With the TMS data collection method described above, Ruit et al. found that the projection algorithm required at least 80 TMS pulses when using the pseudorandom walk method (van de Ruit et al., 2015), and Numssen et al. found that the association algorithm required at least 180 TMS pulses (Numssen et al., 2021). In our study, we also observed the same phenomenon that the association algorithms may require more TMS stimuli than the projection algorithm (Figure 5B, see Supplementary Figure S5B). Interestingly, we found that the EF-COG algorithm consistently performed best, and the MNE algorithm came next, with estimation results mostly showing similarities (Figure 5, see Supplementary Figure S5). The lower performance of the MNE algorithm might be due to the aggravation of the ill-posed problem by decreasing the number of stimuli (Kabanikhin, 2008). Despite working based on points rather than the distribution of the electric field, the max-EF algorithm required a significantly larger N_min_ than the projection algorithm (*p* < 0.001). The instability of the max-EF algorithm may be the reason for its larger N_min_ requirement, as shown in Supplementary Figure S7. The figure illustrates that even if two stimuli induce MEP with a large discrepancy, their maximum electric field cortical sites are very close to each other.

### 4.3 Towards application of clinical and basic neuroscience

TMS motor mapping holds promise in various clinical applications, including pre-surgical planning (Takahashi et al., 2013; Lefaucheur and Picht, 2016), risk stratification (Rosenstock et al., 2017), motor rehabilitation (Lüdemann-Podubecká and Nowak, 2016), as well as basic research such as developmental plasticity (Narayana et al., 2015; Grab et al., 2018; Babwani et al., 2021). The fundamental requirement for a superior mapping algorithm is its ability to accurately delineate the location of the motor cortex. Numerous studies have indicated that the caudal band of the hand area resides in the depth of the central sulcus, and the rostral part is located in the more superficial sulcal wall (Geyer et al., 1996, 2000; Siebner et al., 2022). In this context, the association algorithm stands out as it can capture the entire information of the motor cortex, unlike the projection algorithm, which may miss certain portions (Julkunen, 2014). This suggests that the association algorithm could offer more accurate estimation results, potentially enhancing the security of pre-surgical planning for tumor surgery and providing more detailed knowledge of the motor cortex in research. Consequently, we propose that the association algorithm might be a preferable choice for clinical applications and basic neuroscience research.

TMS serves as a non-invasive technology commonly for causal structure–function mapping through its ability to provide supra-threshold stimulation (Siddiqi et al., 2022). While the objectivity and quantifiability of MEP draw more attention to motor mapping, TMS can extend to mapping cognitive functions beyond motor domains. TMS language mapping is equally significant as a procedure before tumor surgery and is typically conducted using the traditional point-based algorithm (Picht et al., 2013; Babajani-Feremi et al., 2016; Lehtinen et al., 2018). In our study, the association algorithm demonstrated lower prediction error and higher estimation sensitivity in the deep cortical structure. This improvement suggests potential enhancement in the accuracy of TMS language mapping and its applicability to more complex functional mapping. Notably, the association algorithm is utilized in the depression treatment to map the efficacy area of TMS, pending further confirmation regarding the selection of the electric field component (Zhang et al., 2022). Therefore, the association algorithm also exhibits great potential in mapping the efficacy area in the TMS treatment for psychiatry.

### 4.4 Motor mapping divergence between TMS and fMRI

In this study, the group average distance between the location of the cortical hotspot estimated by the projection algorithm and the fMRI activation peak was 16 mm which approached the mean distance reported in previous studies (around 7 to 14 mm) (Herwig et al., 2002; Lotze et al., 2003; Diekhoff et al., 2011). To exclude the possibility that the divergence was caused by the simple projection algorithm itself, we used four other electric-field-based estimation algorithms to re-estimate the cortical hotspot location. The results showed that the divergence in cortical hotspot location (20.52 ± 8.46 mm for five algorithms), as well as in COG (12.21 ± 2.73 mm), remains regardless of the estimation algorithm used (Figure 6, see Supplementary Figure S5). All of the results support the hypothesis that TMS motor mapping differs from fMRI motor task activation mapping.

To further understand the reasons for this divergence, we analyzed the divergence in terms of distance in the 3 axes in MNI space. The notable finding from this analysis is that divergence mainly occurs in the Y axes. The cortical hotspot estimated by projection, max-EF, EF-COG, and MNE algorithms was found to be always significantly more anterior than the fMRI peak, which is consistent with previous studies (Herwig et al., 2002; Lotze et al., 2003; Diekhoff et al., 2011). One possible explanation is that neurons activated by TMS are different from those detected by fMRI (Herwig et al., 2002; Wang et al., 2020). TMS mapping reveals the causal relationship between finger movement and activation of neurons, whereas fMRI mapping simply shows a correlation between the two. Thus the TMS mapping finds neurons that directly cause finger movement and they are mainly in the primary motor cortex (PMC) in the precentral gyrus (Säisänen et al., 2010; Holmes et al., 2019). fMRI mapping detects activation of neurons that are related to voluntary finger movement not only in PMC in the precentral gyrus but also in other regions, such as the somatosensory cortex in the postcentral gyrus (Mima et al., 1999; Ehrsson et al., 2003; Akhlaghi et al., 2012). Another possible explanation is that the brain shift might also result in slightly anterior TMS cortical hotspots. The brain shift results from different conditions during the sMRI when the subject is lying and the TMS session when the subject is sitting. However, we cannot confirm the existence of divergence along the Y-axis yet, as the cortical hotspots estimated by association were not significantly more anterior than the fMRI activation peak (*p* = 0.492). The discrepancy with the association algorithm could be due to its higher estimation accuracy or the insufficient number of participants in our study.

In conclusion, both previous and our own suggest that the deviation between the TMS mapping cortical hotspot and the fMRI activation peak may arise from differences in the neurons activated by TMS compared to those detected by fMRI. Wang et al. also noted that this deviation was linked to distinct brain circuits in non-voluntary and voluntary finger movements (Wang et al., 2020). This deviation suggests the necessity of choosing an appropriate mapping technology based on research objectives. For instance, in the treatment of movement disorder, selecting the fMRI activation peak as the TMS target might be preferable. Considering the deviation between TMS and fMRI mapping, as well as the similarity between TMS and DCS mapping (Coburger et al., 2013; Mangraviti et al., 2013), fMRI mapping could be significantly supplemented by pre-surgical planning to avoid excising the area responsible for voluntary rather than non-voluntary movement.

### 4.5 Limitations and future work

There are several limitations to this study, which will guide our future work. Several enhanced association algorithms have been proposed (Weise et al., 2020; Kataja et al., 2021; Numssen et al., 2021; Weise et al., 2023), with the latest protocol and code for one of them publicly available (Weise et al., 2023). This protocol incorporates additional parameters of the mapping procedure, including I/O curves and coil orientations. This underscores the superiority of algorithms utilizing the electric field modeling, given that the projection algorithm cannot capture the influence of orientations, despite orientation being a crucial parameter in TMS. In our study, we examined five estimation algorithms using a classical motor mapping procedure without regard to the coil orientation. While this facilitated the result of comparisons suitable for the majority of motor mapping scenarios, further investigations employing new motor mapping procedures are needed to demonstrate the superiority of the association algorithm. It was observed that the association algorithm exhibited lower mapping efficiency than the projection algorithm and converged slowly in the second experiment. Further investigation is warranted in the new procedure because the association algorithm might require a more diverse set of TMS pulses to achieve a more reliable mapping.

Although more and more studies strive to demonstrate the physiological significance of the numerical electric field (Argyelan et al., 2019; Jamil et al., 2020; Fridgeirsson et al., 2021; Mosayebi-Samani et al., 2021), the prediction error might not be determined solely by the estimation accuracy of the mapping algorithm. Therefore, in our future work, using the DCS mapping result as the gold standard (Coburger et al., 2011, 2013; Opitz et al., 2014; Seynaeve et al., 2019) is needed for validating the higher estimation accuracy of the association algorithm. Besides the number of stimuli, the reliability of each estimation algorithm is also affected by the combination of stimulation site and orientation. Therefore, future research should explore the optimization of stimulation patterns to enhance the performance of the estimation.

## 5 Conclusion

In this study, we used the same set of experimental data to compare five TMS motor mapping estimation algorithms mainly in two experiments. In the first experiment, we found that the projection algorithm performed best among the point-based algorithms, while the association algorithms performed best among the field algorithms. However, the projection algorithm might miss part of the hand area because it cannot estimate it accurately in the sulcus, and even might not be in the gyrus lip. In the second experiment, we observed that the projection algorithm required fewer stimuli compared to the association algorithms when collecting TMS mapping data using the typical grid-based method. Generally, we suggest that the association algorithm may be a preferable choice for clinical applications and basic neuroscience research, even across various TMS mapping domains, including language area mapping and mapping the areas effective in depression treatment, among others. Finally, we found that even when using advanced estimation algorithms, the location of all cortical hotspots estimated by the five algorithms still deviated from the activation peak obtained from fMRI, without showing a consistent orientation preference.

## Data availability statement

The data analyzed in this study is subject to the following licenses/restrictions: The datasets generated or analyzed during the current study are not publicly available due to the privacy of volunteers but are available from the corresponding author on reasonable request. Requests to access these datasets should be directed to YC 764190303@qq.com.

## Ethics statement

The studies involving humans were approved by the Ethics Committee of the State Key Laboratory of Cognitive Neuroscience and Learning at Beijing Normal University. The studies were conducted in accordance with the local legislation and institutional requirements. The participants provided their written informed consent to participate in this study.

## Author contributions

YC: Conceptualization, Formal analysis, Investigation, Methodology, Validation, Visualization, Writing – original draft, Writing – review & editing. YJ: Data curation, Methodology, Resources, Writing – review & editing. ZZ: Conceptualization, Supervision, Writing – review & editing. ZL: Supervision, Writing – review & editing. CZ: Conceptualization, Funding acquisition, Investigation, Supervision, Writing – review & editing.

## Glossary

TMS: Transcranial magnetic stimulation
MEP: Motor-evoked potential
FDI: First dorsal interosseous muscle
fMRI: functional magnetic resonance imaging
DCS: Direct cortical stimulation
CPC: Continuous proportional coordinate
RMT: Resting motor threshold
I/O curve: Input–output curve
COG: Center-of-gravity
MNE: Minimum norm estimation
N_min_: the minimum number of stimuli needed for stable mapping results
TR: Repetition time
TE: Echo time
FA: Flip angle
FOV: Field of view
EPI: Echo planar imaging
MNI: Montreal Neurological Institute space
