# Supplementary material for: Transcranial magnetic stimulation mapping of the motor cortex: comparison of five estimation algorithms
Source: Front Neurosci. 2023 Dec 7;17:1301075. doi: 10.3389/fnins.2023.1301075 (PMC10733534; doi:10.3389/fnins.2023.1301075)
Supplement: Supplementary file 1 [file Data_Sheet_1.docx]

# Supplement

## Supplementary methods

### Head modeling and electric field simulation

For head modeling, each subject’s high-resolution T1-weighted structural image was acquired on a 332 Siemens Trio 3T MRI Scanner in the Brain Imaging Center at Beijing Normal University, with the following parameters: 176 sagittal slices; repetition time (TR) = 2530; echo time (TE) = 3.5ms; flip angle (FA) = 90°; field of view (FOV) = 256 × 256 mm; slices thickness = 1.0 mm.

The construction of volume conductor model and numerical simulation of the electric field was realized on the SimNIBS v3.2 open-source pipeline [1]. T1 images were segmented into five major tissues (scalp, skull, grey matter, white matter, and cerebrospinal fluid) using cat12 to build a high-solution head mesh. The tissue conductivities were σ_scalp_ = 0.456 S/m, σ_skull_ = 0.01 S/m, σ_GM_ = 0.275 S/m, σ_WM_ = 0.216 S/m, σ_CSF_ = 1.654 S/m [2-3], and all were treated as isotropic. The scalp and cortical surfaces were also reconstructed from T1 images.

The coil positions were offline reconstructed on the scalp surface by measuring the target CPC points according to 4 reference points (nasion, inion, left/right preauricular) [4]. We shifted the coil positions perpendicularly to the scalp surface outward 4mm to compensate for the effect of hair and the swimming cap used for localization. The coil orientations were also reconstructed in the tangent plane of grid points as described in our previous study [5]. The dipole model of the figure-of-eight coil was from Thielscher et al., and it has the same diameter, structure, and manufacturer as the model we used [6], which are all major factors influencing the induced electric field [7-8]. Due to the equal intensity of all stimuli for a subject and the linear relationship between electric field strength and stimulator output, the change rates of the coil current were all set to be 1A/μs. According to the above configurations, the electric potential at each cortical patch was simulated using the finite element model (FEM) solver [9-10].

### Analysis of fMRI data

fMRI Expert Analysis Tool (FSL FEAT, version 6.0, [www.fsl.fmrib.ox.ac.uk/fsl/fslwiki/FEAT](http://www.fsl.fmrib.ox.ac.uk/fsl/fslwiki/FEAT)) was used for the analysis of fMRI data. Preprocessing of raw fMRI scans included: head motion correction (MCFLIRT), high-pass filtering (1/60Hz cutoff), spatial smoothing (4mm Gaussian kernel FWHM), and co-registration to the T1 images (i. e. the partial EPI series was registered onto the whole EPI based on rigid body transformations, and the whole EPI was registered onto the brain extracted from T1 images via the boundary-based registration). A general linear model was used for the subject-level activation analysis of FDI movement. The resulting z-statistical map was corrected at a statistical threshold of P < 0.05 at cluster level using Gaussian random field theory. Then z-statistics were thresholded at 2.3 (corresponding to uncorrected P < 0.01) in each voxel within a given cluster.

## Supplementary results


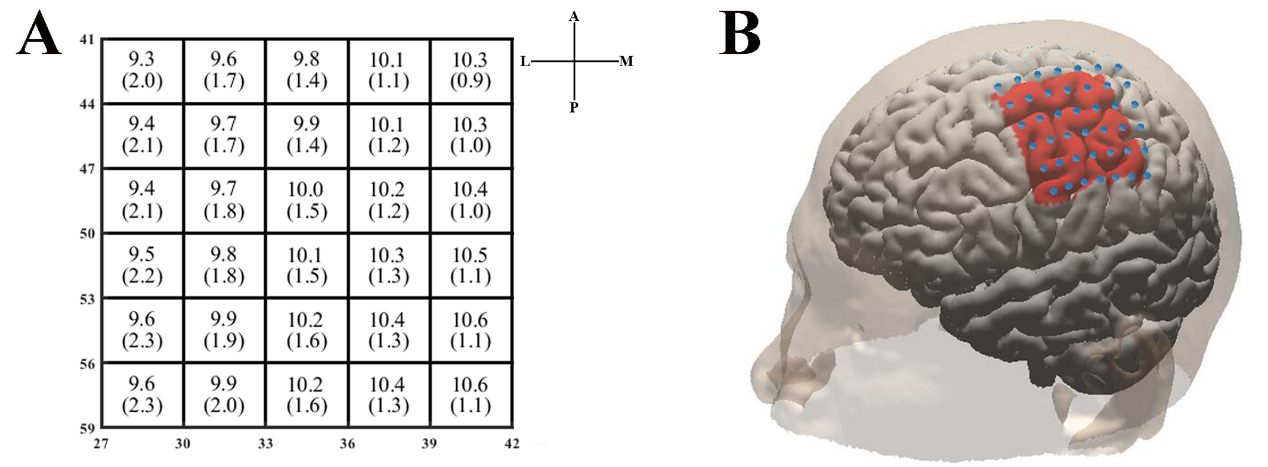


**Fig. S1 Stimulation grid and estimation scope on the cortex.** (A) It's a sketch map of the simulation grid. The number in each grid indicates the group average of mean and standard deviation (in parentheses) of Euclidean distance (mm) of four edges of each grid. Row and column labels are CPC values (rows are Pnz and columns are Pal). A: anterior, P: posterior, L: lateral, M: medial. (B) Example of estimation scope (subject 2) in the red area. Blue points indicate the stimulation grid.


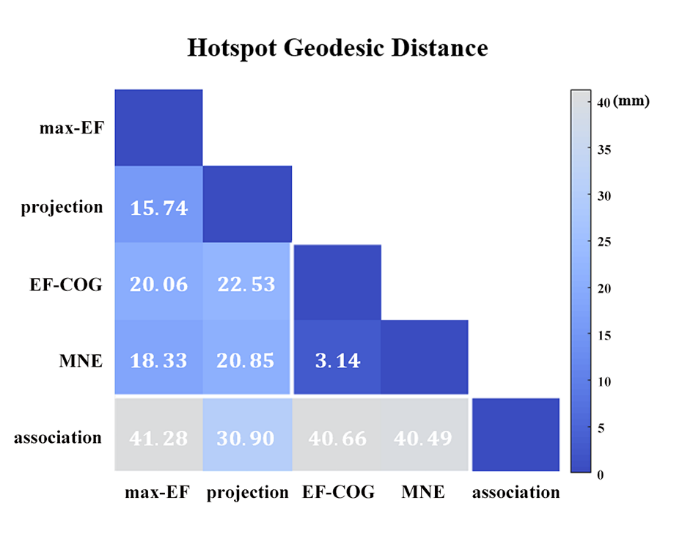


**Fig. S2 Similarity of motor maps from different algorithms.** The similarity in terms of the geodesic distance between hotspots.


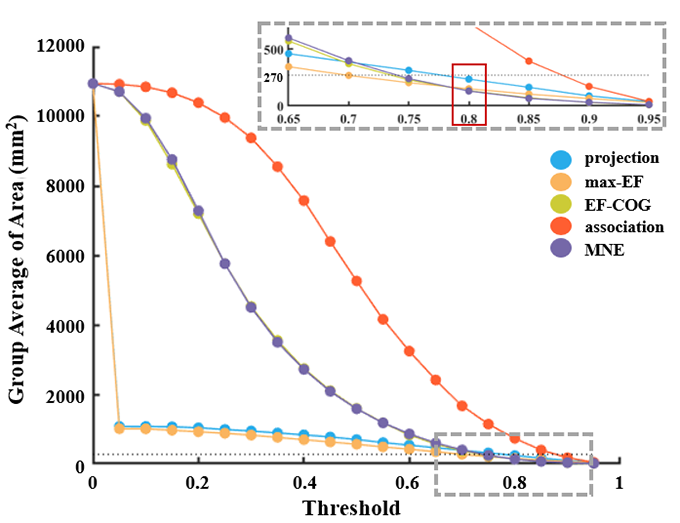


**Fig. S3 The relationship between outlining threshold and group average area of motor cortex estimated by five algorithms.**


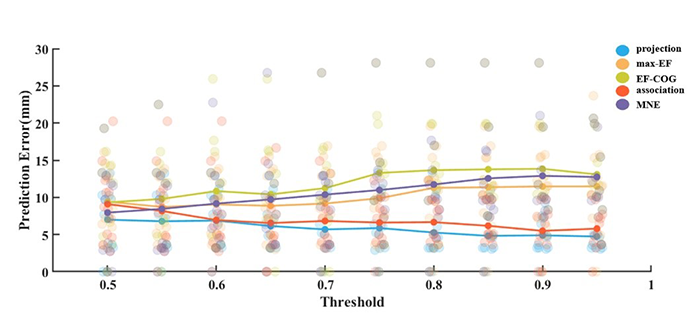


**Fig. S4 The relationship between outlining threshold and prediction error (mm) of five algorithms.** The dot with different colors indicates the prediction of the algorithm represented by the color on one subject.


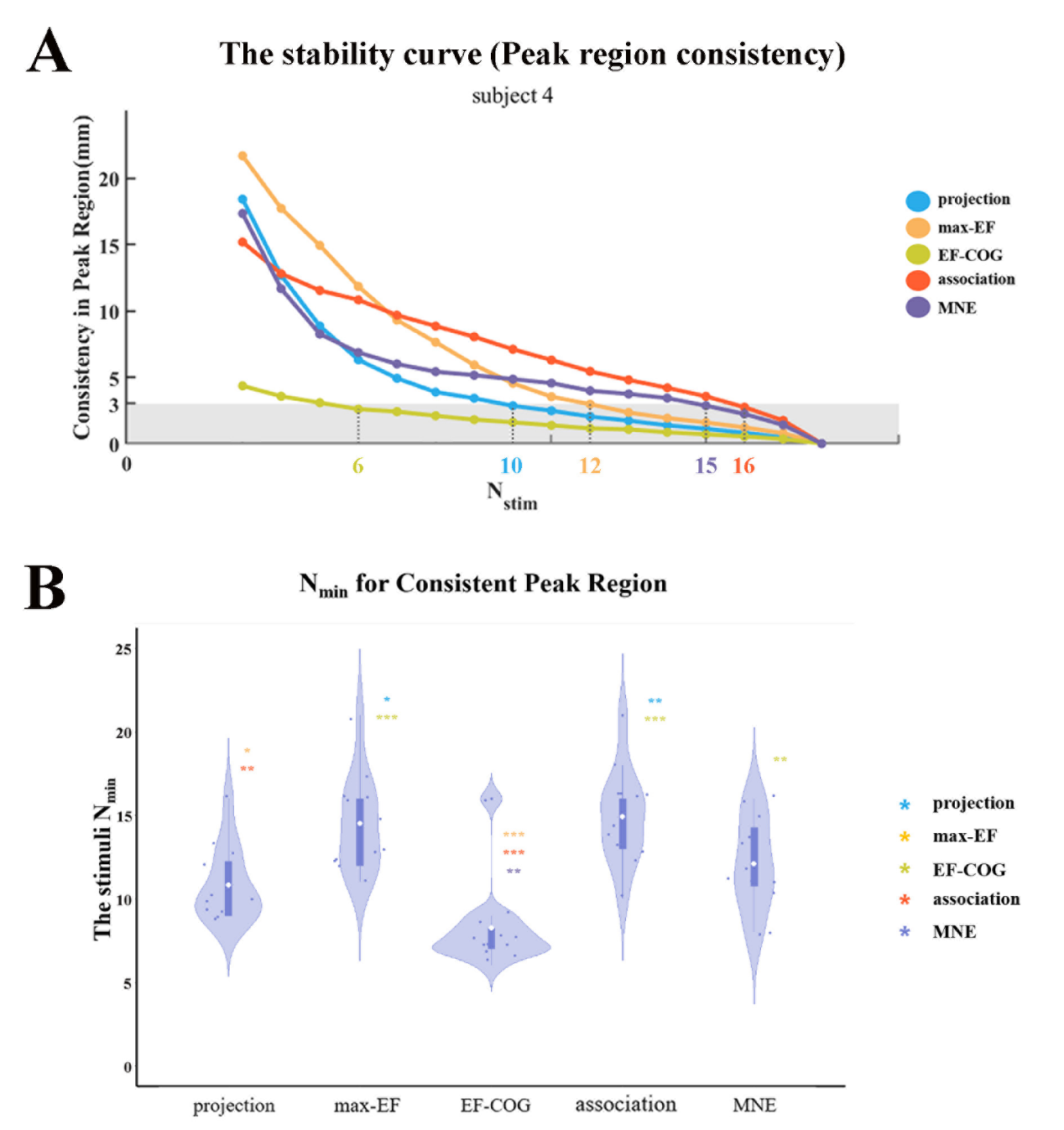


**Fig. S5 The comparison of conservative N_min_ required for reliable estimates.** (A) It shows the relationship between number of stimuli and stability of peak region location of five algorithms. Examples are given for subject 4. Color numbers show N_min_ of the five algorithms, the number of stimuli required for the distance of peak region COG ≤ 3 mm (shade region); (B) Violin plots show the distribution of the N_min_ of the five algorithms. For each algorithm, conservative N_min_ of each subject is represented by the blue dot. White dot represents the group-average N_min_ of each algorithm. Asterisks indicate significant differences between N_min_ of the algorithm plotted and that of another algorithm (represented by different colors). *P<0.05, **P<0.01, ***P<0.001


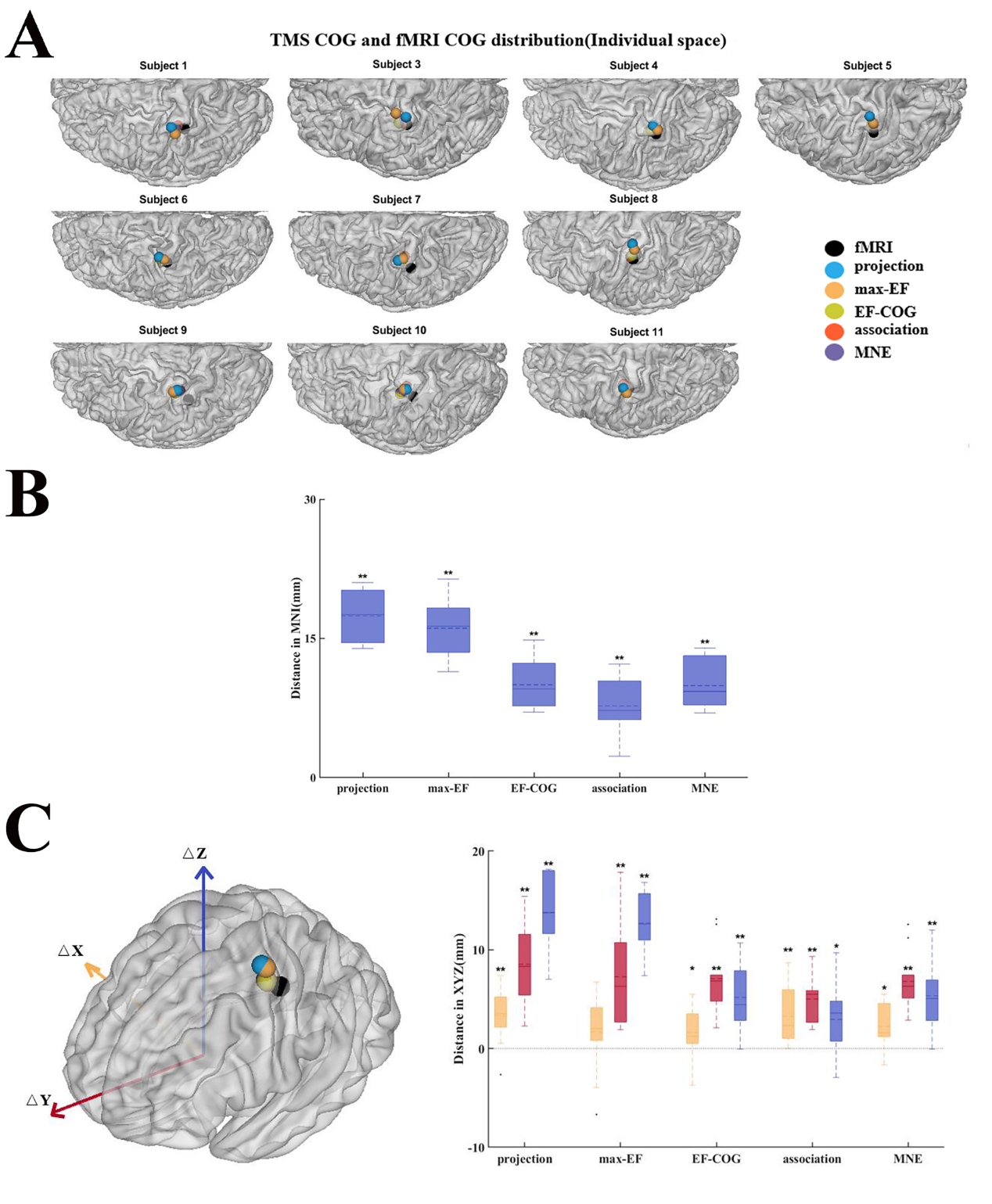


**Fig. S6 Divergence between TMS and fMRI COG.** (A) COGs estimated by the five algorithms and fMRI COG (black sphere) in individual MRI spaces (B) Euclidean distance between TMS COGs and fMRI COG significant differences were found (P = 0.002 for each algorithm). (C) Left panel shows the group-mean COGs estimated by five algorithms and fMRI COGs. Right panel shows the distance in terms of three axes (red, Y-axis; blue, Z-axis; yellow, X-axis). TMS COGs were located significantly more anterior (P=0.002 for each algorithm) and superior to fMRI COG (P_projection_ = 0.002; P_max-EF_ = 0.002; P_EF-COG_ = 0.004; P_association_ = 0.027; P_MNE_ = 0.004). All box plots show median (black solid line), mean (black dashed line), interquartile range (box top and bottom), and 10th and 90th percentiles (error bars). *P<0.05, **P<0.01.


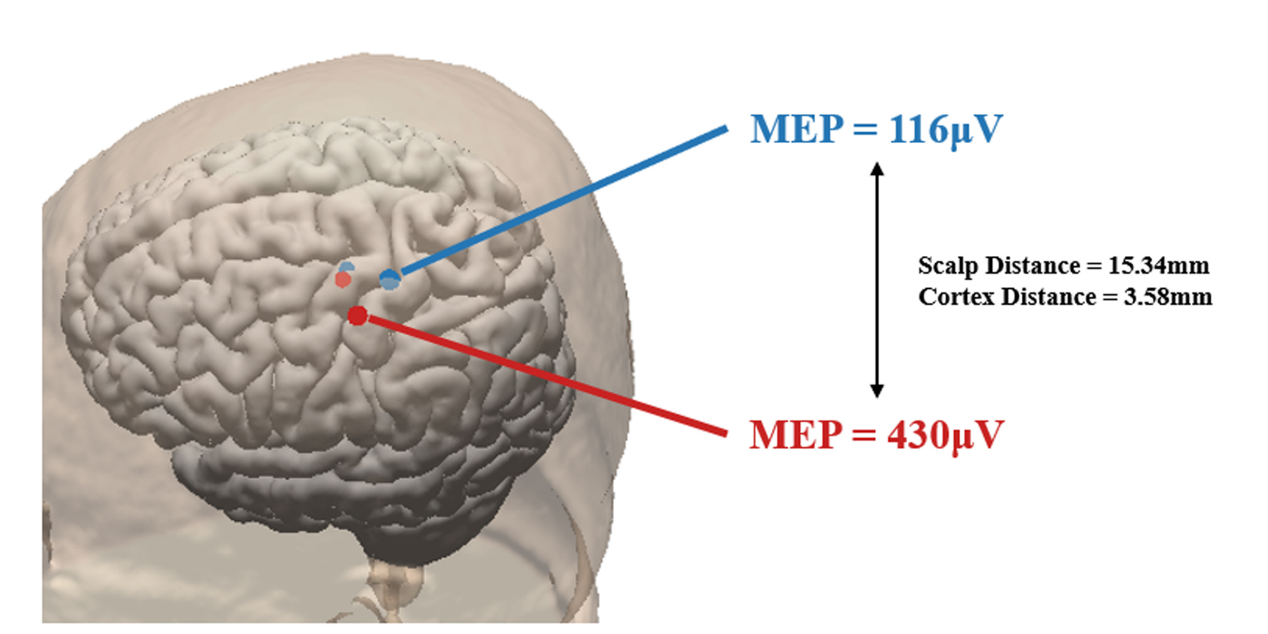


**Fig. S7 Instability of the maximum electric field cortical site.** An example illustrates the instability of the maximum electric field (max-EF) site (subject 1). Two stimulation sites on the scalp (Euclidean distance 15.34 mm) with nearby cortical sites according to max-EF (Euclidean distance is 3.58 mm).

| Time | Number of blocks | Deviation between  TMS and fMRI mapping | Reference |
| --- | --- | --- | --- |
| Movement and rest (37 s) | 6 | Both lay within the omega region | Boroojerdi B, 1999^[11]^ |
| Movement and rest (40 s) | 6 | Average deviation of the hotspot is 13.9 mm | Lotze M, 2003^[12]^ |
| Movement 20 s, rest 40 s | 10 | Average deviation of the hotspot is 10.5-12.8 mm, and that of COG is16.4-18.1 mm | Diekhoff S, 2011^[13]^ |
| Movement 20 s, rest 15 s | 5 | Average deviation of the hotspot is 12.9 mm | Weiss C, 2013^[14]^ |
| Movement 40 s, rest 40 s | 6 | Connected with different network | Wang J, 2019^[15]^ |

**Table S1.** **Block design in fMRI tasks for comparing the TMS and fMRI motor mapping.**

## Supplementary references

1. Thielscher A, Antunes A, Saturnino GB. Field modeling for transcranial magnetic stimulation: A useful tool to understand the physiological effects of TMS? Annu Int Conf IEEE Eng Med Biol Soc. 2015;2015:222-5. doi: 10.1109/EMBC.2015.7318340.
2. Thielscher A, Opitz A, Windhoff M. Impact of the gyral geometry on the electric field induced by transcranial magnetic stimulation. Neuroimage. 2011 Jan 1;54(1):234-43. doi: 10.1016/j.neuroimage.2010.07.061.
3. Wagner TA, Zahn M, Grodzinsky AJ, Pascual-Leone A. Three-dimensional head model simulation of transcranial magnetic stimulation. IEEE Trans Biomed Eng. 2004 Sep;51(9):1586-98. doi: 10.1109/TBME.2004.827925.
4. Xiao X, Yu X, Zhang Z, Zhao Y, Jiang Y, Li Z, Yang Y, Zhu C. Transcranial brain atlas. Sci Adv. 2018 Sep 5;4(9):eaar6904. doi: 10.1126/sciadv.aar6904.
5. Jiang Y, Du B, Chen Y, Wei L, Zhang Z, Cao Z, Xie C, Li Q, Cai Z, Li Z, Zhu C. A scalp-measurement based parameter space: Towards locating TMS coils in a clinically-friendly way. Brain Stimul. 2022 Jul-Aug;15(4):924-926. doi: 10.1016/j.brs.2022.06.001.
6. Thielscher A, Kammer T. Electric field properties of two commercial figure-8 coils in TMS: calculation of focality and efficiency. Clin Neurophysiol. 2004 Jul;115(7):1697-708. doi: 10.1016/j.clinph.2004.02.019.
7. Ueno S, Sekino M. Figure-Eight Coils for Magnetic Stimulation: From Focal Stimulation to Deep Stimulation. Front Hum Neurosci. 2021 Dec 16;15:805971. doi: 10.3389/fnhum.2021.805971.
8. Hedyeh Bagherzadeh and Fow-sen Choa. Effect of coil size on transcranial magnetic stimulation (TMS) focality. Proc. SPIE 11020, Smart Biomedical and Physiological Sensor Technology XV, 110200Z (2 May 2019). doi: 10.1117/12.2524503.
9. Windhoff M, Opitz A, Thielscher A. Electric field calculations in brain stimulation based on finite elements: an optimized processing pipeline for the generation and usage of accurate individual head models. Hum Brain Mapp. 2013 Apr;34(4):923-35. doi: 10.1002/hbm.21479.
10. Saturnino GB, Madsen KH, Thielscher A. Electric field simulations for transcranial brain stimulation using FEM: an efficient implementation and error analysis. J Neural Eng. 2019 Nov 6;16(6):066032. doi: 10.1088/1741-2552/ab41ba.
11. Boroojerdi B, Foltys H, Krings T, Spetzger U, Thron A, Töpper R. Localization of the motor hand area using transcranial magnetic stimulation and functional magnetic resonance imaging. Clin Neurophysiol. 1999 Apr;110(4):699-704. doi: 10.1016/s1388-2457(98)00027-3.
12. Lotze M, Kaethner RJ, Erb M, Cohen LG, Grodd W, Topka H. Comparison of representational maps using functional magnetic resonance imaging and transcranial magnetic stimulation. Clin Neurophysiol. 2003 Feb;114(2):306-12. doi: 10.1016/s1388-2457(02)00380-2.
13. Diekhoff S, Uludağ K, Sparing R, Tittgemeyer M, Cavuşoğlu M, von Cramon DY, Grefkes C. Functional localization in the human brain: Gradient-Echo, Spin-Echo, and arterial spin-labeling fMRI compared with neuronavigated TMS. Hum Brain Mapp. 2011 Mar;32(3):341-57. doi: 10.1002/hbm.21024.
14. Weiss C, Nettekoven C, Rehme AK, Neuschmelting V, Eisenbeis A, Goldbrunner R, Grefkes C. Mapping the hand, foot and face representations in the primary motor cortex - retest reliability of neuronavigated TMS versus functional MRI. Neuroimage. 2013 Feb 1;66:531-42. doi: 10.1016/j.neuroimage.2012.10.046. Epub 2012 Oct 29. PMID: 23116812.
15. Wang J, Meng HJ, Ji GJ, Jing Y, Wang HX, Deng XP, Feng ZJ, Zhao N, Zang YF, Zhang J. Finger Tapping Task Activation vs. TMS Hotspot: Different Locations and Networks. Brain Topogr. 2020 Jan;33(1):123-134. doi: 10.1007/s10548-019-00741-9. Epub 2019 Nov 6.
